# Normative Values of Brainstem Auditory-Evoked Responses in Sheep

**DOI:** 10.3390/brainsci15010069

**Published:** 2025-01-14

**Authors:** Katharina Suntinger, Adrian Dalbert, Lukas Prochazka, Milena Tegelkamp, Peter Kronen, Karina Klein, Christof Röösli, Alexander Huber, Flurin Pfiffner

**Affiliations:** 1 Department of Otorhinolaryngology, Head and Neck Surgery, University Hospital Zurich, University of Zurich, 8006 Zurich, Switzerland; 2 Musculoskeletal Research Unit, Vetsuisse Faculty, University of Zurich, 8057 Zurich, Switzerland; 3 Center for Applied Biotechnology and Molecular Medicine (CABMM), University of Zurich, 8057 Zurich, Switzerland

**Keywords:** auditory-evoked potentials, brainstem auditory-evoked response, otology, ovine animal model, sheep

## Abstract

**Background:** The brainstem auditory-evoked response (BAER) is an established electrophysiological measure of neural activity from the auditory nerve up to the brain stem. The BAER is used to diagnose abnormalities in auditory pathways and in neurophysiological human and animal research. However, normative data for BAERs in sheep, which represent an adequate large animal model for translational and basic otological research, are lacking. **Objective:** The aim of this study was to assess the function of the ovine auditory nervous system by determining normative values for the BAER and to compare sheep with human BAER data. **Methods:** In this retrospective study, BAER data for click stimuli at a range of sound pressure levels (SPLs) were analyzed. A series of 15 samples from six sheep with a mean age of 41.8 months was included. **Results:** The mean BAER threshold was 45.3 dB SPL. At 100 dB SPL, the mean (±standard deviation, SD) latency of wave V was 4.35 (±0.18) ms, that of wave III was 2.44 (±0.15) ms, and that of wave I was 0.88 (±0.13) ms. At 100 dB SPL, the mean interpeak latency of waves I–III was 1.56 (±0.18) ms, that of waves III–V was 1.91 (±0.16) ms, and that of waves I–V was 3.47 (±0.20) ms. The mean amplitudes at 100 dB SPL were 0.04 (±0.03) µV for wave I, 0.50 (±0.24) µV for wave III, and 0.40 (±0.25) µV for wave V. **Conclusions:** The normative values for sheep BAERs were reproducible and similar to those of humans. The normative BAER values further support sheep as an adequate animal model for otological research.

## 1. Introduction

Since the first reports of the clinical application of brainstem auditory-evoked responses (BAERs) in the mid-1970s, BAER assessment has become a standard, well-established electrophysiological method for investigating the state of the cochlea, auditory nerve, and central auditory pathway within the midportion of the brainstem [1]. Currently, the BAER has a broad scope of interdisciplinary applications; for example, it is clinically utilized in neurology and audiology as well as in pediatric and intensive care settings. Furthermore, BAER assessments in animals serve as a basis for animal research and contribute to the understanding of the function and organization of the auditory nervous system.

Animal models in otology (a branch of medicine that studies normal and pathological anatomy and physiology of the ear) have become increasingly important for the development and exploration of surgical techniques and medical devices such as implantable hearing aid systems. Common animal models for research in otology include gerbils, cats, guinea pigs, and chinchillas. However, because of the significantly different anatomical proportions and frequency bandwidth of hearing in non-human animals compared to humans, these small animals have limited applicability as models for research into human hearing.

Similarities in the ascending human auditory nervous system, including central auditory processing, have shown that primates are an appropriate animal model for such research. However, ethical constraints and the requirement for special facilities limit their use as animal models. Because of the similarities in anatomy, histology, and physiology between the hearing organs of sheep and humans [2], sheep are increasingly considered to be suitable large animal models for otological studies [3]. In addition, as it is possible to use human-sized surgical equipment and implants, recent studies have used sheep as a model animal. Examples of similar anatomical functions between humans and sheep include the middle ear [4], round window [5], and cochlea [6]. Furthermore, sheep temporal bones have been successfully used as surgical training models [7], for surgical ossicular chain reconstruction [8], and cochlear implantation [6]. Sheep have also been adequately used as models for endoscopic ear surgery [9], for the stenting of the Eustachian tube [10], to deliver therapeutics to the inner ear [11], and for the development of implantable hearing devices [12].

To the best of our knowledge, the most recent sheep BAER data were published in 2020, in a study suggesting the potential use of BAER for the detection of cochlear trauma during cochlear implant insertion [13]. Two previous studies investigated pathology-correlated changes in BAER in sheep with anemia [14] or scrapie [15]. Other studies primarily investigated BAER in fetal and newborn sheep [16], providing physiological data from ewes as a reference cohort. However, varying values of BAERs in sheep have been reported.

A conclusive homogeneous database of BAER data are a requirement for in vivo studies that use sheep as an animal model. This study aimed to create a set of normative values for BAERs in Swiss White Alpine sheep to provide a basis for future in vivo clinical trials evaluating the state of and changes in the auditory pathway within the brainstem.

## 2. Methods

### 2.1. Animal Enrollment

In this retrospective analysis, data from two studies were included, which were conducted in accordance with the Swiss Regulations of Animal Welfare and were approved by local federal authorities (code ZH 136/16 and code ZH 105/18). The BAERs of 6 female Swiss White Alpine sheep, which yielded 15 BAER recordings for 12 ears, were analyzed (Table 1).

Unilateral recordings of sheep (S1), with two follow-up measurements, and a single unilateral recording of S2 were included. Bilateral data from 3 sheep (S3, S4, and S5) were used, with a unilateral follow-up after approximately 2 months. Additionally, a unilateral recording of S6 during the first assessment and another unilateral recording from the opposite side during the second assessment were used. The mean age of the included sheep was 41.8 months (range 35–56 months), and the mean weight before the initial measurement was 80.4 kg (range 72.4–91.5 kg).

### 2.2. BAER Assessments

The BAER assessments were performed on anesthetized sheep in an operating room at a veterinary university hospital in Zurich. After orotracheal intubation, the sheep were positioned in sternal recumbency on an operating table. Perioperative antibiotic prophylaxis with amoxicillin/clavulanic acid (Synulox^®^, Zoetis GmbH, Delémont, Switzerland) or amoxicillin (Duphamox^®^, Zoetis GmbH, Delémont, Switzerland) was administered. All drugs were administered via central intravenous line placed in the jugular vein. For induction buprenorphine (0.01 mg/kgBW), medetomidine (0.007 mg/kgBW), ketamine (mean 3.0 mg/kgBW, range: 2.95–3.04), midazolam (0.1 mg/kgBW), and propofol (mean 0.76 mg/kgBW, range: 0.37–1.37) were administered. For yjr maintenance of general anesthesia, ketamine (mean 1.15 mg/kgBW/h, range: 0.51–1.26) and propofol (mean 2.1 mg/kgBW/h, range: 1.89–3.78) were used, both being administered via constant rate infusion. Body temperature was monitored during anesthesia (mean 38.1 °C, range 37.5–38.9 °C, at the end of the anesthesia procedure). Additionally, an orogastric tube was inserted for the drainage of excess gastric fluid. Postoperatively, all sheep were kept on the veterinary campus until the completion of follow-up measurements.

First, the external ear canal was inspected and cleaned of cerumen and dirt. An insert earphone (580-BINSER, Bio-logic Systems Corp., Mundelein, IL, USA) and a probe reference microphone (ER-7C, Etymotic, Inc., Elk Grove Village, IL, USA) were placed via a foam tip in the ear canal. The sound pressure level (SPL) recorded by the reference microphone was monitored on a digital oscilloscope (Type RTB2004, Rohde & Schwarz, Munich, Germany) to control the SPL near the tympanic membrane. The insert foam earphone was replaced if a difference of more than 5 dB was detected between the chosen acoustic stimulation level in the BAER measurement software (software version 7.0.0, Biologic Systems Corp., Mundelein, IL, USA) and the corresponding level measured with the reference microphone in the ear canal.

The acoustic stimuli were clicks with alternating phases. The stimulation rate was 13.3 clicks/s, with a stimulus duration of 100 μs. The acoustic stimulation level, beginning at 100 dB SPL, was decreased in 20 dB steps until no BAER signal was visually identified. BAER recordings were then obtained in 10 dB steps near the level of the last recording with visually confirmed responses. The BAER threshold level was defined as the lowest stimulation SPL with an identifiable wave V.

BAER recordings were performed with the Bio-logic Navigator Pro device and Auditory Evoked Potentials (AEP) software, version 7.0.0 (Biologic Systems, Mundelein, IL, USA). After shaving and cleaning the respective lambskin area, disposable needle electrodes (20 × 0.3 mm, Neurosign, Magstim Co., Wales, UK) were placed on the ipsilateral retroauricular region (“positive”), in the contralateral retroauricular region (“negative”), and on the forehead (“ground”). The retroauricular electrode placement was on the mastoid plane, lateroinferiorly in the area of the posterior cartilaginous base of the ear. The neutral electrode was placed in the area of the sheep’s nasal bone, approximately midway between the orbit and the nasal process of the nasal bone. The electrical impedances of all recording locations were less than 6 kΩ. The BAERs were averaged over 2000 repetitions. Continuous averaging was stopped before 2000 repetitions only if the displayed AEP response was clearly visible and remained constant after 1000 repetitions. The recording window was 10.66 ms, starting 0.8 ms before stimulus presentation. The recording amplifier’s low-pass filter was set at 3000 Hz, and the high-pass filter at 70 Hz. A 23.80 μV threshold was selected for artifact rejection. The gain of the preamplifier was set to 100,000. For the three bilateral measurements (S3, S4, S5), two-channel recording montages were used and recorded by the two channels of the BAER recording device.

AEP to ASCII software version 7.0.0 from Biologic Systems was used to export the data from the AEP software database. The data were analyzed and plotted using GraphPad Prism V5.04 (GraphPad Software Inc., La Jolla, CA, USA). The collected waveforms were analyzed independently by three experienced clinicians and included the assessment of the amplitudes and latencies of waves I, II III, IV, and V. Absolute latency was defined as the time between the acoustic stimulus and the positive wave peak. The interpeak latencies of I–III, III–V, and I–V were derived from the absolute latencies. Amplitudes were measured from peak to trough (against polarity) because of large inter-individual differences in absolute amplitude values. For a comparison of the recordings, the means and standard deviations (SDs) for absolute latencies, interpeak latencies, and peak-to-trough amplitudes were determined at several sound pressure levels (100, 80, 60, and 40 dB SPL).

## 3. Results

In this retrospective study, 15 available BAER recordings of 12 ears of 6 sheep were analyzed.

### 3.1. Sample Data

**Figure 1:**
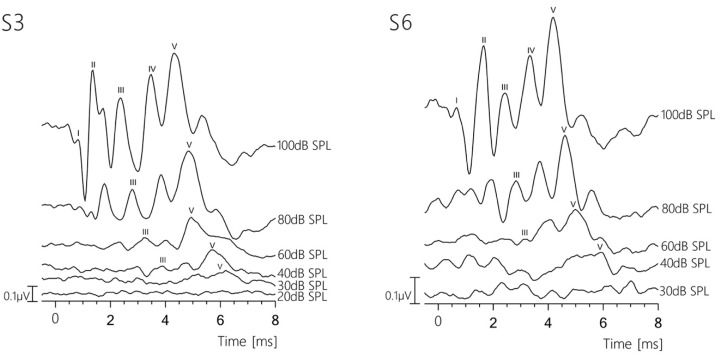
Two representative brainstem auditory-evoked response recordings from sheep S3 (**left**) and S6 (**right**) at descending SPLs. The *X*-axis represents the time window between −0.5 ms and 8 ms, and the reference bar indicates amplitude values in µV. The five wave amplitudes are labeled I, II, III, IV, and VI.

Figure 1 shows two representative BAER recordings of sheep S3 and S6 at different stimulation levels. Sheep S3 had a BAER threshold level of 30 dB SPL, while that of S6 was 40 dB SPL. At 80 dB SPL, the latencies of waves III and V were 2.61 and 4.82 for S3, and 2.95 and 4.99 for S6.

### 3.2. Absolute Latencies

The mean latencies and standard deviations at descending stimulation levels for all BAER recordings are summarized in Table 2. At 100 dB SPL, the mean latency of wave V was 4.35 (±0.18) ms, that of wave III was 2.44 (±0.15) ms, and that of wave I was 0.88 (±0.13) ms. The threshold level of click-evoked BAERs (visible in wave V) was 40 dB SPL for 11 recordings and 60 dB SPL for 4 of the 15 recordings. The overall range of wave V was 0.58 ms at 100 dB SPL, SPL 0.82 ms at 80 dB, SPL 1 ms at 60 dB, and 0.92 ms at 40 dB SPL. The range of other waves over all SPLs was similar.

**Figure 2:**
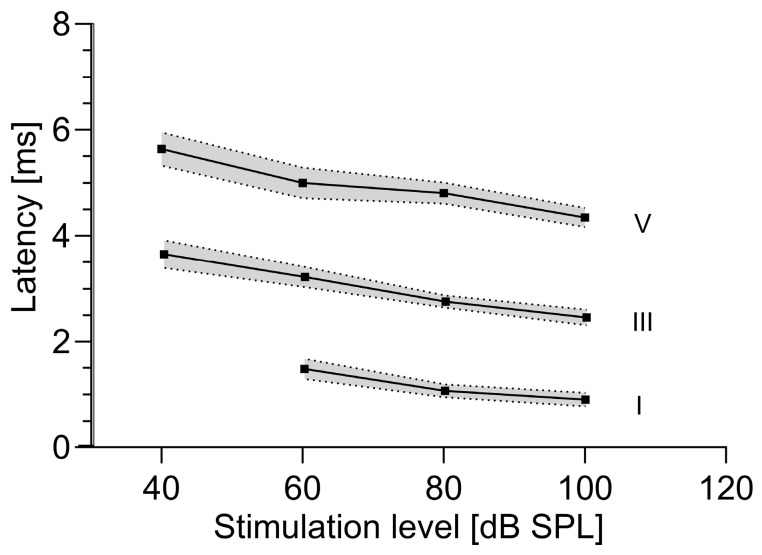
Decreasing latency of the most prominent waves with increasing stimulus for all brainstem auditory-evoked response recordings. The latency (*Y*-axis) is depicted as a function of the stimulation level (*X*-axis). The black squares mark the mean values, and the gray shaded areas indicate the standard deviations.

Figure 2 displays the latencies of the most prominent waves, i.e., waves I, III, and V, at decreasing stimuli for all BAER recordings. As shown in Table 2, at a stimulation level of 40 dB SPL, not all BAER waves (I to V) could be identified in all 15 recordings (11 BAERs showed wave V, 8 showed wave III, and wave I was not visible in any recording).

**Figure 3:**
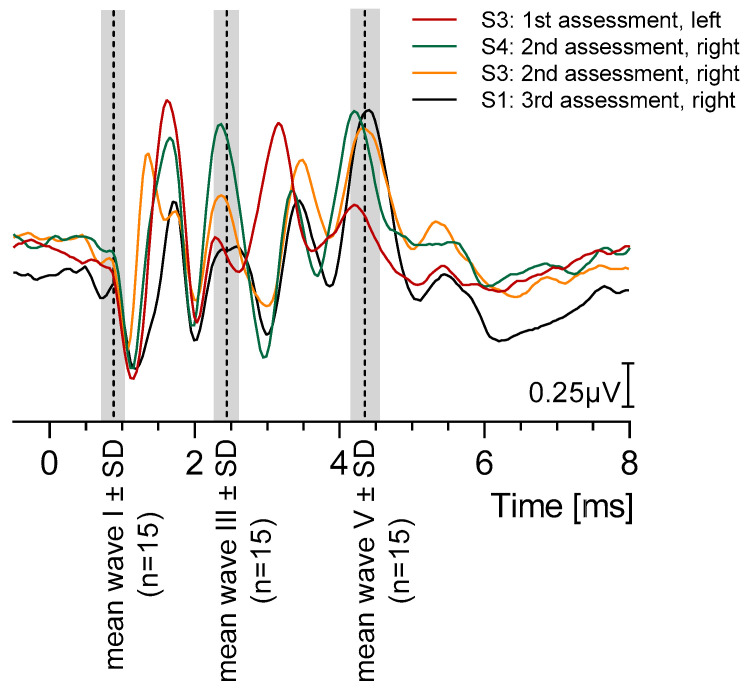
Four representative brainstem auditory-evoked response recordings at 100 dB SPL. The overall wave-specific mean latencies of waves I, III, and V are marked by a dashed vertical line. The SD of each peak is indicated as a gray bar.

Four representative BAER recordings at 100 dB SPL stimulation level are shown in Figure 3.

### 3.3. Interpeak Latencies

The BAER interpeak latencies at acoustic stimulus levels of 100, 80, and 60 dB SPL are summarized in Table 3. The mean interpeak latencies of waves I–III were between 1.56 ms (100 dB SPL) and 1.76 ms (60 dB SPL), those of waves III–V were between 1.79 ms (60 dB SPL) and 2.06 ms (80 dB SPL), and those of waves I–V were between 3.47 ms (100 dB SPL) and 3.76 ms (80 dB SPL). While the absolute mean latencies decreased with an increasing stimulus (e.g., for wave V from 5.00 ms at 60 dB SPL to 4.35 ms at 100 dB SPL), the interpeak latencies showed lower relative change (0.2 ms for wave I–III, 0.27 ms for wave III–V, and 0.29 ms for I–V) and no distinct dependence on the stimulation level.

**Figure 4:**
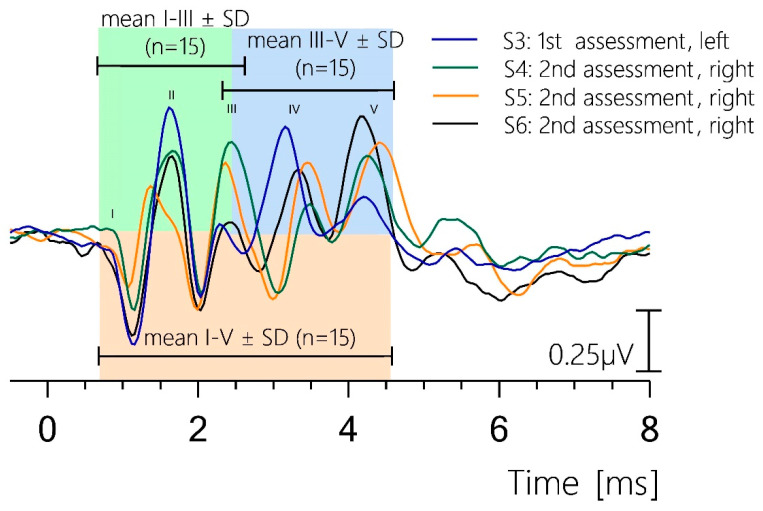
Representative brainstem auditory-evoked response recordings at 100 dB SPL. Absolute amplitudes of waves I, III, and V ranged from 0.026 to 0.84 µV (peak-to-trough measurement). Mean interpeak latencies were 1.56 ms for waves I–III (green), 1.91 ms for waves III–V (blue), and 3.47 ms for waves I–V (orange). The corresponding SD values are displayed with a horizontal black bar (SD for waves I–III latency: 0.18, for waves III–V: 0.16, and for waves I–V: 0.20).

Figure 4 shows four representative BAER recordings from different subjects at 100 dB SPL stimulus. The absolute amplitudes of waves I, III, and V ranged from 0.026 to 0.84 µV when measured peak-to-trough.

### 3.4. Peak-to-Trough Amplitudes

Table 4 summarizes the mean and SD peak-to-trough amplitudes of all BAER recordings. At lower stimulation levels (60 and 40 dB SPL), the wave V had the largest peak-to-trough amplitude values (mean 0.16 and 0.10 µV) compared to the other waves.

**Figure 5:**
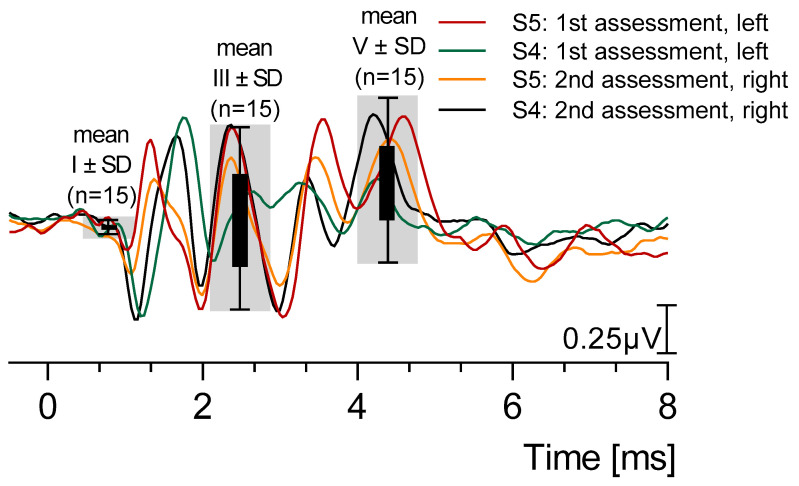
Four representative recordings of the brainstem auditory-evoked responses (BAERs) of sheep S4 and S5 at 100 dB SPL. The overall mean amplitude with standard deviation (SD) of waves I (mean 0.04 µV, SD 0.03), III (mean 0.50 µV, SD 0.24), and V (mean 0.40 µV, SD 0.25) are indicated by the gray bars.

Four representative recordings of the BAERs of S3 and S4 at 100 dB SPL are shown in Figure 5. The overall mean amplitude with SD of waves I (mean 0.04 µV, SD 0.03), III (mean 0.50 µV, SD 0.24), and V (mean 0.40 µV, SD 0.25) are indicated by the gray bars.

### 3.5. Reproducibility

**Figure 6:**
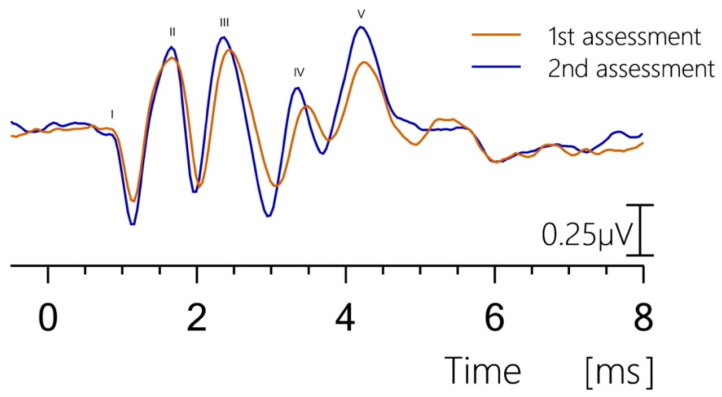
Reproducibility of brainstem auditory-evoked response (BAER) assessments in a representative recording. BAER assessment (sheep S4 at 100 dB SPL) was repeated 64 days after the first assessment. A good visual agreement between the two measurements can be stated (blue and red curves, respectively).

An important criterion for validating the recordings was the reproducibility of the BAER. This was verified with follow-up measurements for certain cases (*n* = 5). The reproducibility of the BAER measurements was determined from the variation in the absolute and the interpeak latencies between measurements taken in the same subject on different days. Figure 6 shows an example for such repeated BAER assessments in the same subject. The waveforms (S4 at 100 dB SPL) show good qualitative agreement between the two 64-day displaced measurements (red and blue curves, respectively). Table 5 summarizes the mean variations in wave latencies (wave I, III, and V) between follow-up assessments at 100 dB SPL and the five subjects considered. A mean absolute latency variation between 0.12 and 0.18 ms and a mean absolute interpeak latency between 0.13 and 0.29 ms indicates satisfactory reproducibility.

## 4. Discussion

Sheep are a suitable large animal model with increasing importance in otology. However, the normative values for BAERs, an effective objective measurement method for the characterization of hearing at different time intervals during clinical trials, were lacking for Swiss White Alpine sheep. Here, we demonstrated the feasibility of BAER measurements in sheep and provided a set of normative values for in vivo sheep experiments. With these normative values, the real-time evaluation of the auditory pathway can be performed during clinical trials using ovine models.

The normative values of the absolute latencies of waves I and V and the interpeak latencies are particularly useful for assessing the auditory function of sheep during BAER measurements. A real-time comparison of recorded BAER data can be made with the reference data presented in Table 2 and Figure 3.

While the sample size (*n* = 9 ears) is modest, which is a limitation of this study, the reproducibility of the BAER data was confirmed through repeated recordings (Figure 6 and Table 5). Despite the reproducible data obtained in this study, previous reports of BAER in healthy adult sheep provided contradictory information about wave morphology as well as the presence of individual waves and latencies, particularly for waves IV and V [17,18].

The latency of wave V has been reported at around 6 ms at 93 dB(A) [19], approximately 4.7 ms at 90 dB (Figure 3, [14]), and 3.35–3.87 ms at 95 dB nHL [15]. DeYoung and colleagues [18] reported a wave IV/V complex with a latency of 5.12 ms at 105 dB SPL. Kaufmann and colleagues reported the mean latency of wave V as 7 ms at 110–115 dB SPL. It may also be hypothesized that these latency prolongations could be the result of differences in the stimuli (burst stimuli instead of clicks). In comparison to the wide range of wave V latencies reported in the literature (3.35–7 ms), our data produced wave V latencies near the lower end of the range reported by others (4.35 ms at 100 dB SPL to 4.80 ms at 80 dB SPL; Table 2).

Most of the waves persisting at lower stimulus levels with a deep trough before wave VI were identified as wave V (Figure 1, Figure 3, Figure 4, Figure 5 and Figure 6). Griffiths et al. (1996) [16] reported the latency of wave IV in sheep as equivalent to wave V in humans due to a merged wave IV-V-complex in sheep, which they concluded to often result in different wave identifications. In addition, Wilson and Mills (2005) [20] reported an often-absent wave IV in dogs, resulting in the questionable labeling of wave V. DeYoung and colleagues [18] reported a wave IV–V complex that was sometimes rather difficult to identify. Our BAER recordings from sheep exhibited a shape similar to human BAERs, as shown in the representative recordings in Figure 1, Figure 3, Figure 4, Figure 5 and Figure 6. When the stimulus levels were near the threshold, waves IV and V merged into a complex in some cases; however, both peaks were clearly identifiable as two separate peaks at higher stimulus levels. This can be observed in the recording of S6 at 60 dB SPL in Figure 1.

The same research group that reported the absence of wave V in sheep measured latencies of wave IV of 4.02 ms (adult) and 4.03 ms (lambs, 7 weeks old) at 41 dB nHL [16,21]. As shown in Table 2, wave IV appeared with lower mean latencies of 3.4 ms at 100 dB SPL and 3.7 ms at 80 dB SPL in our study.

Konold et al. (2016) [15] presented the largest ovine BAER dataset to date (*N* = 141). While the mean latencies of waves I, II, and III (at 95 dB nHL: 1.00–1.25 ms, 1.80–2.10 ms, and 2.50–3.05 ms, respectively) were comparable to our data (at 80 dB SPL: 1.04 ms, 1.85 ms, and 2.74 ms, respectively), the presence of a wave IV was not reported. The latencies of wave IV (mean 3.74 ms) in our study might be equivalent to those of wave V (3.35–3.87 ms) in the study of Konold et al. (2016) [15]. Although the wave latencies in our study and those of Konold et al. seem comparable, it is difficult to make any conclusions on the differentiation of IV and V waves.

Further discrepancy between the studies might result from the fact that Konold et al. (2016) [15] analyzed a heterogeneous group of sheep with regard to sex (81 females, 7 entire males, 53 neutered males), age (median age: 51 months, range 17–111 months), and breed (67 Suffolk, 48 Cheviot, 22 Romney, and 4 Poll Dorset sheep). In our study, we considered a homogeneous group of sheep to exclude sex- (female sheep have shorter latencies [15]), age- (age of the included animals ranges from 35 to 56 months), and the expected breed dependency of the BAERs.

Also, other studies included more heterogeneous groups of sheep of different ages and breeds, such as *Ovis aries* [22], bighorn sheep [17], Suffolk [23,24], Suffolk/Dorset crossbreed [13], and *Ovis canadensis* [18], or did not report the breed at all [16,25]. In addition, there is no consistent reporting of the drugs used, age, sex, body temperature, and weight of the animals. Thus, the potential effects of these intrinsic parameters on the BAER cannot be determined.

However, the previously discussed variability in BAER latencies might be due to differences in breeds such as, for example, different sizes, weights, or anatomical features. Similar studies with dogs indicate relevant effects of maturation [26,27,28], head size [23,29], and breed [30]. This suggests a potential limitation in the generalizability of our results to other sheep breeds.

Apart from the breed, age, and sex of the animals, additional reasons for the differences in wave morphology and wave latencies might be attributed to the placement and type of the recording electrodes and the subjective visual identification of the waves. Exogenous influences, such as the configuration of the BAER measurement setup with different acoustic stimuli, as well as varying stimulation rate and stimulation level, further limit comparisons among studies.

One challenge in this study was to ensure the correct acoustic stimulation level in the ear canal. Because of the narrower and more S-shaped ear canal of sheep compared to humans, the insertion of the earphone can lead to lower acoustic levels. Furthermore, several insertion trials were needed to maintain a sufficiently high stimulation level in the ear canal. Falsified stimulation pressure measurements in the ear canal attributed to inadequate earphone placement may explain why other studies have reported longer latencies than those in our data. This might also be a reason for the lower percentage of identifiable BAER recordings (down to 40%) [13]. Reference microphone placement near the tympanic membrane ensures correct acoustic stimulation and the specification of the stimulus level in dB SPL, instead of in units related to normal human hearing (e.g., nHL), facilitates comparison between studies.

The core question when using animal models is the applicability and generalizability of the findings to humans. Table 6 summarizes normative human BAER ranges of absolute and interpeak latencies for normal hearing subjects reported by Mauerer [31] and Hood [32].

The mean absolute wave latencies for sheep with acoustic stimulation at 80 dB SPL were 1.04 ms for waves I, 2.74 ms for waves III, and 4.80 ms for waves V, showing shorter latencies compared to ranges in humans reported by Mauerer [31] and Hood [32]. The highest deviation (around 1.1 ms) showed wave III. The mean sheep interpeak latencies of waves III–V (2.06 ms) and I–V (3.76 ms) are close to the reported human range. The mean sheep interpeak latencies of waves I–III are below the reported human values, mainly because of the shorter absolute latency of wave III in sheep compared to humans.

## 5. Conclusions

Sheep are a valuable animal model in otology because of their similarities to humans in terms of auditory anatomy and physiology, as well as for economic reasons. The normative values presented here provide a basis for the interpretation of in vivo BAER results in sheep.

## Figures and Tables

**Table 1:** Overview of BAER recordings.

Sheep No.	BAER Recorded Ear [Left/Right/Bilateral]			Included Recordings [*n*]	Time Between Assessments [Days]
	First Assessment	Second Assessment	Third Assessment		
S1 *	right	right	right	3	3/4 *
S2	right	n/a	n/a	1	n/a
S3	bilateral	right	n/a	3	65
S4	bilateral	right	n/a	3	64
S5	bilateral	right	n/a	3	64
S6	left	right	n/a	2	63
total	9 (right: 5/left: 4)	5	1	*N* = 15	

* S1: A third assessment was performed on the right side; the time between the second and third assessments was 4 days. n/a: not applicable.

**Table 2:** Mean latencies of waves I–V at different sound pressure levels (SPLs).

Intensity [dB SPL]	Number of BAERs	Mean Latency (and Standard Deviation) [ms]				
		Wave I	Wave II	Wave III	Wave IV	Wave V
100	15	0.88 (0.13)	1.62 (0.15)	2.44 (0.15)	3.36 (0.15)	4.35 (0.18)
80	15	1.04 (0.13)	1.85 (0.13)	2.74 (0.12)	3.74 (0.15)	4.80 (0.20)
60	15	1.45 (0.19)	2.40 (0.13)	3.21 (0.19)	4.02 (0.22)	5.00 (0.29)
40	0/2/8/8/11	n.i.	2.95 (0.06)	3.65 (0.26)	4.50 (0.18)	5.64 (0.31)

BAERs: brainstem auditory-evoked responses; n.i.: not identifiable.

**Table 3:** Mean interpeak latencies of waves I–V at different sound pressure levels.

Sound Pressure Level [dB SPL]	Number of BAERs	Mean Interpeak Latency [ms] and SD		
		I–III (±SD)	III–V (±SD)	I–V (±SD)
100	15	1.56 (0.18)	1.91 (0.16)	3.47 (0.20)
80	15	1.70 (0.10)	2.06 (0.19)	3.76 (0.26)
60	15	1.76 (0.20)	1.79 (0.20)	3.54 (0.30)

**Table 4:** Mean amplitudes (peak-to-trough) of waves I–V at different sound pressure levels.

Sound Pressure Level [dB SPL]	Subjects [*n*]	Mean Amplitude [µV] + SD									
		I	±SD	II	±SD	III	±SD	IV	±SD	V	±SD
100	15	0.04	0.03	0.84	0.46	0.50	0.24	0.63	0.28	0.40	0.25
80	15	0.04	0.02	0.19	0.13	0.23	0.15	0.29	0.15	0.22	0.14
60	15	0.03	0.02	0.05	0.06	0.07	0.03	0.09	0.06	0.16	0.09
40	0/2/8/8/11	n.i.	n.i.	0.08	0.08	0.06	0.02	0.07	0.03	0.10	0.05

SD: standard deviation; n.i.: not identifiable.

**Table 5:** Mean variations in latencies of I, III, and V waves between follow-up assessments at 100 dB SPL.

Latency	Subjects [*n*]	Mean Difference [ms] ± SD	
		Mean [ms]	±SD
I–III	5	0.29	0.18
III–V	5	0.13	0.10
I–V	5	0.29	0.17
I	5	0.18	0.11
III	5	0.12	0.10
V	5	0.16	0.12

SD: standard deviation.

**Table 6:** Comparison of mean latencies of waves I–V in humans [31,32] and sheep.

Stimulation Level	Subject	Mean Latency (and Standard Deviation) and Interpeak Latency in [ms]					
		Wave I (ms)	Wave III (ms)	Wave V (ms)	Wave I–III (ms)	III–V (ms)	I–V (ms)
80 dB SPL	Sheep	1.04 (0.13)	2.74 (0.12)	4.80 (0.20)	1.70 (0.10)	2.06 (0.19)	3.76 (0.26)
70 dB HL	Human [31]	1.56 (0.12)	3.83 (0.2)	5.76 (0.22)	2.27	1.93	4.20
80 dBnHL	Human [32]	1.37–1.93	3.44–4.16	5.18–6.10	1.87–2.43	1.56–2.12	3.59–4.39

## Data Availability

The raw data supporting the conclusions of this article will be made available by the authors on request.
